# Supplementary material for: Modeling glioblastoma heterogeneity as a dynamic network of cell states
Source: Mol Syst Biol. 2021 Sep 16;17(9):e10105. doi: 10.15252/msb.202010105 (PMC8444284; doi:10.15252/msb.202010105)
Supplement: Supplementary file 6 — Source Data for Figure 5 [file MSB-17-e10105-s004.zip › Figure5A_sourcedata/GSEA_3017/nosigdb_stateD.GseaPreranked.1622795010397/FELDMAN_2020_LATEG1.html]

Details for gene set FELDMAN\_2020\_LATEG1[GSEA]

|  || Dataset | state23017 |
| Phenotype | NoPhenotypeAvailable |
| Upregulated in class | na\_neg |
| GeneSet | FELDMAN\_2020\_LATEG1 |
| Enrichment Score (ES) | -0.56089735 |
| Normalized Enrichment Score (NES) | -2.6541135 |
| Nominal p-value | 0.0 |
| FDR q-value | 0.0 |
| FWER p-Value | 0.0 |
Table: GSEA Results Summary

  

Fig 1: Enrichment plot: FELDMAN\_2020\_LATEG1      
 Profile of the Running ES Score & Positions of GeneSet Members on the Rank Ordered List

  

| PROBE | GENE SYMBOL | GENE\_TITLE | RANK IN GENE LIST | RANK METRIC SCORE | RUNNING ES | CORE ENRICHMENT || 1 | PTTG1 |  |  | 14 | 0.490 | 0.0062 | No |
| 2 | ARL4C |  |  | 24 | 0.422 | 0.0140 | No |
| 3 | SQLE |  |  | 25 | 0.421 | 0.0288 | No |
| 4 | IDI1 |  |  | 34 | 0.382 | 0.0359 | No |
| 5 | RAB13 |  |  | 43 | 0.367 | 0.0425 | No |
| 6 | TGIF1 |  |  | 55 | 0.343 | 0.0459 | No |
| 7 | PHLDA1 |  |  | 59 | 0.336 | 0.0554 | No |
| 8 | MYC |  |  | 77 | 0.313 | 0.0531 | No |
| 9 | HMGB3 |  |  | 80 | 0.311 | 0.0624 | No |
| 10 | SOX4 |  |  | 91 | 0.298 | 0.0650 | No |
| 11 | BIRC5 |  |  | 130 | 0.271 | 0.0447 | No |
| 12 | MKI67 |  |  | 206 | -0.254 | -0.0052 | No |
| 13 | SORBS2 |  |  | 318 | -0.265 | -0.0830 | No |
| 14 | PRSS23 |  |  | 498 | -0.284 | -0.2135 | No |
| 15 | CYR61 |  |  | 555 | -0.292 | -0.2473 | No |
| 16 | PRC1 |  |  | 645 | -0.310 | -0.3062 | No |
| 17 | S100B |  |  | 705 | -0.320 | -0.3413 | No |
| 18 | CDC20 |  |  | 793 | -0.341 | -0.3976 | No |
| 19 | MAD2L1 |  |  | 819 | -0.350 | -0.4049 | No |
| 20 | MEST |  |  | 864 | -0.360 | -0.4268 | No |
| 21 | CKAP2 |  |  | 900 | -0.375 | -0.4411 | No |
| 22 | TPX2 |  |  | 1034 | -0.437 | -0.5302 | No |
| 23 | PLK2 |  |  | 1047 | -0.444 | -0.5240 | No |
| 24 | ARID5B |  |  | 1095 | -0.467 | -0.5445 | Yes |
| 25 | SAT1 |  |  | 1109 | -0.473 | -0.5381 | Yes |
| 26 | ARHGAP29 |  |  | 1121 | -0.487 | -0.5296 | Yes |
| 27 | DDAH1 |  |  | 1132 | -0.497 | -0.5200 | Yes |
| 28 | NUSAP1 |  |  | 1154 | -0.515 | -0.5184 | Yes |
| 29 | CNN3 |  |  | 1181 | -0.552 | -0.5194 | Yes |
| 30 | CDCA3 |  |  | 1187 | -0.563 | -0.5035 | Yes |
| 31 | ASPM |  |  | 1189 | -0.567 | -0.4844 | Yes |
| 32 | CKS1B |  |  | 1198 | -0.577 | -0.4704 | Yes |
| 33 | BEX1 |  |  | 1216 | -0.615 | -0.4621 | Yes |
| 34 | SERPINE1 |  |  | 1218 | -0.617 | -0.4412 | Yes |
| 35 | ANXA1 |  |  | 1221 | -0.623 | -0.4209 | Yes |
| 36 | SMC4 |  |  | 1224 | -0.638 | -0.4001 | Yes |
| 37 | CKS2 |  |  | 1226 | -0.641 | -0.3783 | Yes |
| 38 | CTGF |  |  | 1234 | -0.654 | -0.3608 | Yes |
| 39 | CENPA |  |  | 1243 | -0.679 | -0.3433 | Yes |
| 40 | CCNA2 |  |  | 1255 | -0.716 | -0.3267 | Yes |
| 41 | HMGB2 |  |  | 1263 | -0.736 | -0.3064 | Yes |
| 42 | ID3 |  |  | 1276 | -0.785 | -0.2882 | Yes |
| 43 | CDK1 |  |  | 1311 | -1.433 | -0.2645 | Yes |
| 44 | UBE2C |  |  | 1312 | -1.466 | -0.2130 | Yes |
| 45 | TOP2A |  |  | 1313 | -1.528 | -0.1594 | Yes |
| 46 | CCL2 |  |  | 1317 | -1.880 | -0.0957 | Yes |
| 47 | HIST1H4C |  |  | 1320 | -2.769 | -0.0000 | Yes |
Table: GSEA details [plain text format]

  

Fig 2: FELDMAN\_2020\_LATEG1: Random ES distribution      
 Gene set null distribution of ES for **FELDMAN\_2020\_LATEG1**
